# Fundamental Stability Skills: Reliability Analysis Using the *Alfamov* Assessment Tool

**DOI:** 10.3390/children11050583

**Published:** 2024-05-11

**Authors:** Eva Santos-Miranda, Aida Carballo-Fazanes, Ezequiel Rey, Inés Piñeiro-García-Tuñón, Cristian Abelairas-Gómez

**Affiliations:** 1 CLINURSID Research Group, Universidade de Santiago de Compostela, 15782 Santiago de Compostela, Spain; eva.santos.miranda@sergas.es (E.S.-M.); aida.carballo.fazanes@usc.es (A.C.-F.); 2 Simulation, Life Support, and Intensive Care Research Unit (SICRUS), Health Research Institute of Santiago de Compostela (IDIS), 15706 Santiago de Compostela, Spain; 3 Centro de Saúde de Vedra, Servizo Galego de Saúde, 15885 Vedra, Spain; 4 Faculty of Nursing, Universidade de Santiago de Compostela, 15782 Santiago de Compostela, Spain; 5 REMOSS Research Group, Faculty of Education and Sport Sciences, Universidade de Vigo, 36005 Pontevedra, Spain; zequirey@uvigo.gal; 6 Faculty of Education Sciences, Universidade de Santiago de Compostela, 15782 Santiago de Compostela, Spain; ines.pineiro.garciatunon@rai.usc.es

**Keywords:** fundamental movement skills, stability skills, agreement, assessment, raters, test–retest reliability, intrarater reliability, interrater reliability

## Abstract

Fundamental movement skills (FMS), considered as building blocks of movement, have received growing interest due to their significant impact on both present and future health. FMS are categorized into locomotor, object control and stability skills. While there has been extensive research on assessing the proficiency and reliability of locomotor and object control skills, stability skills have received comparatively less attention. For this reason, this study aimed to assess the test–retest, intrarater and interrater reliability of five stability skills included in the *Alfamov* app. The performance of eighty-four healthy primary school children (60.8% girls), aged 6 to 12 years (mean ± standard deviation of 8.7 ± 1.8 years), in five stability skills was evaluated and scored by four raters, including two experts and two novices. The *Alfamov* tool, integrating various process-oriented tests, was used for the assessment. Reliability analyses were conducted through the computation of the intraclass correlation coefficient (ICC) along with the corresponding 95% confidence intervals. Good-to-excellent intrarater reliability, excellent interrater reliability and moderate-to-good reliability in the test–retest were achieved. The results proved that *Alfamov* is a robust test for evaluating stability skills and can be suitable for use by different professionals with less experience in assessing children’s motor competence.

## 1. Introduction

Fundamental movement skills (FMS) are described as the fundamental building blocks of movement that enable the efficient performance of physical tasks, as they serve as the basis for routing the performance of more complex movements [1]. According to Corlett and Mandigo [2], FMS constitute the first level of physical mastery necessary for the attainment of physical literacy (PL), which is described as the “motivation, confidence, physical competence, knowledge, and understanding to value and take responsibility for engagement in physical activity across the lifespan” [3,4]. Although the multiple benefits of physical activity practice in the prevention of noncommunicable diseases and in the acquisition of healthy lifestyles are well known [5,6,7,8], a large proportion of the world’s population does not reach the desired levels, either due to lack of time, lack of ability or lack of enjoyment, rather than structural difficulties [9].

The study of FMS has shown relevant data regarding its association with health in the short, medium and long term, since its development is related not only to an active lifestyle, but also to an increase in the social, emotional and cognitive well-being of the individual [10,11]. Specifically, the range between 6 and 12 years of age, according to some authors, is the most sensitive stage for the development of FMS [12]. That is why the proper development of these skills in childhood is essential to improve physical, mental and social health [13,14] as they influence these positive or negative trajectories, acquired in the most vulnerable periods, in the medium and long term [15].

There are several classifications of FMS, depending on a variety of criteria [16]. One of the most widely accepted and used is the one described by Gallahue et al. [17] which divides FMS into three subgroups: locomotor skills, which are those that allow the translation of the body in space, such as running, jumping or galloping; manipulative skills, which involve the control and/or manipulation of objects, such as hitting, kicking, throwing or catching; and stability skills, such as rolling or balancing, which are those that allow maintaining postural stability during static or dynamic movement [17,18]. Although this classification places stability skills at the same level as the other two subgroups, they have been described as the most basic skills within this FMS classification model, and have even been referred to as an inherent part during the realization of locomotor and object control skills [17]. Rudd et al. [19] justified the importance of placing stability skills as a separate subgroup, because their development could not reach their full potential if focused exclusively on the other two subgroups.

Given the importance of FMS competence in immediate and future health together with the secular decline that is occurring in children’s motor competence levels [20,21,22], both their promotion and the monitoring and assessment of motor competence are considered crucial. Accordingly, a recent systematic review has identified 57 motor competence assessment tools, but there remains a lack of agreement on which is the most appropriate in a given context [23]. Tests for assessing FMS are primarily categorized into two types: process-oriented assessments, which assess movement quality (e.g., the Victorian HMB Assessment Instrument [24]), and product-oriented assessments, which assess the outcomes (e.g., the Athletic Skills Track [25,26]). Although there is no consensus on which type of instrument to use, some experts opt for process-oriented assessments, as they provide more detailed information on the development of each skill, although more time is required to put them into practice [27].

FMS assessment is hampered by aspects such as culture, lack of knowledge of the tools by the teachers, or the time required to carry out the assessment [28,29,30]. In addition, tracking motor competence across the lifespan is difficult due to limitations of the assessment tools themselves, such as lack of feasibility, lack of accountability for the importance of what is being assessed, limited sensitivity, or limitations of adaptability, so further efforts are needed to develop assessment strategies that have a positive effect on the usability of health outcomes [23].

That is why it is necessary to analyze the validity and reliability of the assessment tools to be used [31] so that their use can guide us in the early detection of delays or disorders in the development of FMS, to perform a close follow-up and make appropriate decisions in order to enhance the capabilities of each individual [32]. Locomotor and object control skills have been analyzed more extensively, since one of the most widely used process-oriented tests for the evaluation of FMS is the Test of Gross Motor Development (TGMD) by Ulrich [33,34], a test that includes the locomotor and object control subscales [1,22]. Other examples of the most widely used batteries such as the AST (product-oriented test) [25] and the Victorian FMS Assessment Instrument (process-oriented test) [24] also do not include the stability skills assessment. For this reason, reliability data for the stability subscale are limited, as very few batteries include them [35,36,37,38].

In the context of the search for a tool to facilitate the assessment of FMS, *Alfamov* was created in 2018, a computer application designed to assess the motor competence of children between 6 and 12 years old through the assessment of 22 skills encompassed in the three subscales: locomotor skills, manipulative skills and stability skills. Three test batteries were used for its creation: the Test of Gross Motor Development (version 2 and 3), Get Skilled: Get Active and the Victorian FMS Assessment [39]. Specifically, the stability skills included were static balance [37], line or beam walk, forward roll [40], rock and log rolling [19]. Against this background, this study attempts to contribute to addressing the pressing need for reliable assessment tools to assess stability fundamental motor skills. For this reason, the aim of the present study was to evaluate the test–retest, intra- and interrater reliability of the five stability skills included in the *Alfamov* app. We hypothesize that *Alfamov* will show at least moderate-to-excellent test–retest, intra- and interrater reliability.

## 2. Materials and Methods

### 2.1. Participants and Study Design

Eighty-four healthy primary school children (comprising 60.7% girls) between 6 and 12 years old actively participated in this cross-sectional study conducted at a public school in Santiago de Compostela, Spain. The inclusion criteria were age, attending the chosen schools, having signed the informed consent form and not having any physical impairment that impeded them from completing the test.

Prior to their involvement, signed informed consent was acquired from the children’s parents or guardians, supplemented by verbal assent from each child. In addition, all children were informed that they could leave this study at any time if they considered it convenient. The data collected during the project were meticulously recorded in a coded and aggregated format. Adherence to national regulations and adherence to ethical principles outlined in the Declaration of Helsinki were strictly followed in this study. The study protocol received approval from the Faculty of Education and Sport Sciences at the University of Vigo.

### 2.2. Raters

Four raters (convenience sample), were involved in assessing of five stability skills performed by participating children. Among them, two were experts in motor competence assessment—one nurse (Rater A) and the other held a BSc in Physical Education and Sports Science (Rater B). The remaining two, who were novices, were primary school teachers (Raters C and D). All assessors were selected based on their integral role in promoting and assessing children’s motor development. Prior the coding process, each novice rater thoroughly reviewed the manual containing the assessment criteria (Table 1) and conducted a practice assessment with a minimum of three children. During this training, they also had the chance to pose questions to the expert raters.

### 2.3. Instruments—Alfamov

*Alfamov* is a process-oriented battery developed for assessing young children’s (aged 6–12 years) gross motor skill performance. Comprising 22 FMS—encompassing 10 locomotor skills, 7 manipulative skills, and 5 stability skills—*Alfamov* is the result of a comprehensive review of scientific literature involving various assessment tools [19,37,40]. It is structured into two main areas:

(1) Didactic Section: Providing comprehensive information on the FMS, their potential significance, detailed insights into each skill, and a demonstration video illustrating the correct execution of each skill.
(2) FMS Assessment Section: This segment allows for real-time assessments (capturing a child performing the skill) or deferred evaluations (choosing a previously recorded video from the device’s gallery).

*Alfamov* supplies a checklist for the assessments featuring performance criteria for each skill, scored by raters. Criteria are rated “1” for met or “0” for not met. The cumulative performance criteria for each skill determine its score. Each skill is assessed twice. Furthermore, *Alfamov* presents subscale scores (locomotor, manipulative and stability) by aggregating results from the two attempts of all the component skills. Ultimately, it provides an overall motor competence score. Table 1 provides a description of stability skills, associated performance criteria and the maximum scores attainable for each skill. The performance criteria were extracted from the batteries mentioned and reviewed by experts, with consensus being reached on those that generated the most doubts as they were more subjective [41].

### 2.4. Procedure

The administration of stability skills included in *Alfamov* adhered to manuals’ guidelines [19,37,40] and was conducted by a study researcher who received specific training for the task. Data collection was carried out during the months of February to March 2023.

**Figure 1:**
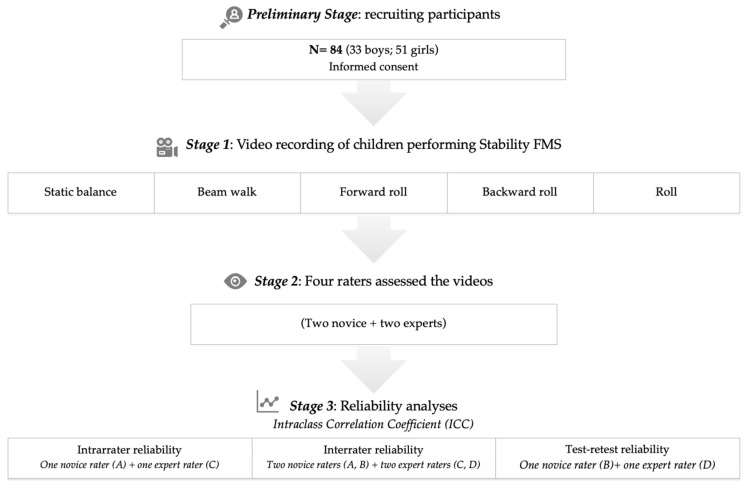
Flowchart of the study design.

Figure 1 provides a summary of the study flow chart. Each child was instructed in the skills through a verbal explanation of the task coupled with a practical demonstration. Subsequently, school children executed a practice trial, followed by two recorded trials (Sony DCR-SR52E) for later scoring (stage 1). One camera was positioned laterally or frontal, depending on the skill, attempting to ensure a correct angle that permitted the vision of all body movements. The assessment took place in a sports hall, during the school’s regular schedule and always in the presence of at least one physical education teacher from the school. The testing procedure depended on the age of the children ranging from 15 to 30 min.

The four raters conducted independent assessments of the videos of the 84 participants (stage 2) to analyze interrater reliability (stage 3). In no cases have there been cross-reporting of the assessments carried out by the raters. In addition, at least 15 days after the initial assessment, two raters (one novice and one expert), conducted a secondary analysis of the children’s performance for intrarater reliability. In order to examine the test–retest reliability, the school children were assessed twice within 10–15 days by two raters (one novice and one expert). During all evaluations, raters were granted the flexibility to watch the videos as many times as needed, with the option of interruptions and slow-motion playback if required.

### 2.5. Statistical Analyses

Reliability analyses were conducted through the computation of the intraclass correlation coefficient (ICC) along with the corresponding 95% confidence intervals (CIs). Consistent with the approach outlined by Koo and Li [42], a two-way mixed-effects model of single measurement and absolute agreement was employed for assessing both test–retest and intrarater reliability. In case of interrater reliability, single measurement, consistency, and two-way mixed-effects model were employed. Values < 0.50 indicated poor reliability, values between 0.50 and 0.75 indicated moderate reliability, values between 0.75 and 0.90 indicated good reliability, and values > 0.90 indicated excellent reliability. All analyses were performed using the SPSS statistical package version 23 (SPSS Inc., Chicago, IL, USA). A significance level of *p* < 0.05 was considered.

## 3. Results

Intrarater, interrater and test–retest reliability results are presented below. This study involved testing a total of 84 schoolchildren aged between 6 and 12 years old (mean ± standard deviation of 8.7 ± 1.8 years). However, only 61 participants were able to attempt the backward roll and 74 participants in the case of forward roll, due to developmental immaturity or fear in the remaining participants.

### 3.1. Intrarater Reliability

Intrarater reliability results (ICC and 95%) are shown in Table 2. Intrarater reliability has been analyzed for two different raters, one expert (Rater A) and one novice (Rater C). ICCs ranged from moderate-to-good reliability for roll (Rater A) and beam walk (Rater A) to excellent for static balance (Rater C). Both raters obtained good-to-excellent reliability in forward roll and backward roll. In the overall computation, for both novice and expert raters, excellent reliability was achieved.

### 3.2. Interrater Reliability

Table 3 shows the interrater reliabilities (ICC and 95% CI). The four raters were involved in the interrater reliability analysis. All skills had good-to-excellent reliability values. Interrater reliability was good-to-excellent for the five skills. The stability overall score indicated excellent interrater reliability with an ICC ranging between 0.907 and 0.954.

### 3.3. Test–Retest Reliability

Table 4 shows scores and test–retest reliability for stability skills (assessed by two raters, one expert [Rater B] and one novice [Rater C]). The overall score indicates moderate-to-good test–retest reliability.

The reliability analysis of each skill individually revealed poor-to-moderate ICC values for both raters in terms of static balance, roll and beam walk. The remaining skills (forward roll and backward roll) obtained moderate-to-good results for both novice and expert raters.

## 4. Discussion

The aim of this study was to assess the interrater, intrarater and test–retest reliability of five fundamental movement stability skills included in the *Alfamov* app, including static balance, beam walk, forward roll, backward roll and roll. For this purpose, four raters, two experts and two novices, were selected to evaluate these skills in 84 schoolchildren. Reliability studies of FMS have primarily focused on assessing locomotor and object control, with most assessment batteries not considering stability skills [23]. However, stability skills were found to be an independent factor in an FMS model and should therefore be assessed separately from the other skills [19].

In terms of intrarater reliability, the existing literature on stability FMS is particularly limited compared to studies focusing on interrater and test–retest reliability. Moreover, the previous studies examining intrarater reliability have involved expert raters [43,44,45], whereas our study analyzed two raters, one novice and one expert. Considering that the purpose is the implementation and use of the motor competence assessment batteries by teachers in charge of elementary schoolchildren, our study considered the novice factor that these professionals may have when using batteries to assess reliability.

In this sense, excellent overall scores were observed in the case of the novice evaluator and good-to-excellent in the case of the expert evaluator for intrarater reliability, coinciding with other publications in which intrarater reliability for stability skills in process-oriented batteries was very high [23]. In other studies, in which locomotor and manipulative FMS were evaluated, significant differences can be observed between novice and expert raters, ensuring that evaluations performed by expert raters or those with experience in physical education obtained more consistent data [46,47,48]. Thus, prior training or consensus was urged before using these batteries, in order to increase their consistency, as differences between raters were recently observed in process-oriented tests [49]. They have objectified the lack of consensus when interpreting and categorizing the scores obtained in the reliability studies, making the objective comparison of the scores obtained in these studies complicated. Therefore, while one rater may interpret the result of his reliability analysis as poor, another may interpret it as good or acceptable, and this can be extrapolated to the test–retest reliability studies. In comparison to our study, we observed no such differences, and the results seem to be consistent regardless of the subjective nature of the process-oriented tests. In comparison to our study, we did not observe such differences, indicating that prior experience in evaluating FMS does not seem to be a determining factor when assessing stability skills using *Alfamov*. Considering that the primary users of these batteries are often teachers and physical education professionals who may not have previous experience with this type of batteries [50], this can be a positive aspect of *Alfamov*.

Regarding the scores obtained in the interrater reliability analysis, the overall results were excellent. Findings in terms of individual skills show good-to-excellent reliability, similar to Rudd et al. [19], for the roll and backward roll skills. It should be noted that, despite using a process-oriented assessment test, we achieved higher results compared to other studies that assessed locomotor and manipulative skills and obtained considerably lower scores [47,48,49,50,51]. This could be attributed to the fact that participants in our study used for assessment not only the performance criteria for each skill, but also the clarifications on those skills considered more subjective in a previous study in which expert raters reached an agreement, as recommended by Carballo-Fazanes et al. [41].

In this sense, similar results to ours were found, in the Eddy et al. [52] study, where they assessed the interrater reliability of the static balance and beam walk in children with motor difficulties. Although they used the Kappa statistic to measure the difference between raters, the total scores showed substantial levels of agreement, with an “almost-perfect” interrater reliability.

In the test–retest reliability analysis, a moderate-to-good overall score was achieved in our study. In comparison, Rudd et al. [19] obtained excellent results in the roll and forward roll skills as expressed by the Kappa statistic. However, in our study, we achieved low scores, with moderate-to-good ICC values for the backward roll and poor-to-moderate for the roll, similar to those obtained by Hulteen et al. [23] in the test–retest analysis carried out with another process-oriented battery, the Maastrichtse Motoriek Test. On the other hand, in other study that assessed the static balance and the beam walk, both skills achieved excellent test–retest reliability scores, which were significantly higher than the poor-to-moderate reliability scores obtained in our study [53]. The reliability data in the last study [52] were reflected in the absolute value of its ICC, without taking into account the confidence intervals, which may explain why they obtained higher values than in our study. Although the values obtained vary for each skill, the overall score is moderate-to-good as previously mentioned, which is consistent with similar test–retest studies assessed in locomotor and manipulative skills using statistical descriptors similar to ours [43,44,54,55].

The low test–retest reliability values do not appear to be related to the raters’ experience, since similar scores have been achieved in almost all the skills, as well as in the overall score. We hypothesize that these results could be attributed to the fact that the schoolchildren participants have had enough time to practice and improve these skills between the two assessment days carried out in the test–retest, since practically all the motor competence skill scores were higher in the second assessment.

Furthermore, in a recent study by Hulteen et al. [49], where different interrater reliability studies of the TGMD were analyzed and compared, they have objectified the lack of consensus when interpreting and categorizing the scores obtained in the reliability studies, making the objective comparison of the scores obtained in these studies complicated. Therefore, while one author may interpret the result of his reliability analysis as poor, another may interpret it as good or acceptable, and this can be extrapolated to test–retest reliability studies.

When contrasting the results obtained in all the reliability assessments, one of the main problems encountered is the lack of specific reliability research for the FMS of stability. The fact that for some authors these skills are an intrinsic part of the development of the skills included in the subgroups of locomotor and manipulative skills limits the literature in this regard [17]. Since these skills are an important aspect of FMS, we must recognize the limitations of using batteries that do not evaluate them, and taking into account that some of the most widely used batteries do not include the stability skills subgroup, such TGMD [24,25,33,34], added to the lack of consensus in the interpretation of the values of the reliability studies [49], makes it more difficult to compare the reliability stability results.

### Limitations

This study is not free from limitations. A total of four raters were used in our study, which, although considered an adequate number for reliability studies, was a convenience sample without random selection. For this reason, the results should be interpreted with caution and it would be advisable to repeat this study with different raters. On the other hand, in terms of general methodological improvements needed in this area of research, the use of different statistical analyses in different studies makes it difficult to compare reliability results and it would be desirable to standardize this approach.

## 5. Conclusions

This study assessed the intrarater, interrater and test–retest reliability of the five stability FMS included in the *Alfamov* app. The results showed good-to-excellent intrarater and excellent interrater reliability and a moderate-to-good reliability in the test–retest. Furthermore, the reliability proved to be high regardless of the raters’ experience, which could indicate that *Alfamov* is a robust test and can be suitable for use by different professionals with less experience in assessing children’s motor competence.

## Figures and Tables

**Table 1:** Description of stability skills included in *Alfamov*.

	*Material* Procedure/Instruction	Performance Criteria *[Consensus]*	Maximum Score [T1 + T2]
Static balance ^1^	-- Maintaining balance on one leg for as long as possible.	1. Support leg still, foot flat on the ground.	10
		2. Non-support leg bent, not touching the support leg. *[If it is touched on one occasion, it is scored 0.]*	
		3. Eyes focused forward.	
		4. Head and trunk stable and upright.	
		5. Arms extended without excessive movement.	
Line or beam walk ^2^	*A line of 5 m drawn on the ground.* Walk slowly along the line, ensuring both feet remain on it.	1. Walks fluidly without pauses.	8
		2. Keeps both feet on the beam or line with toes facing the front. *[The foot must be in line with the line. If the heel touches, but the toe does not, it is 0. If the foot is parallel to the line, but not completely on the line, 1.]*	
		3. Head and trunk stable and facing the front.	
		4. Uses arms when necessary to maintain balance. *[Both arms separate from the trunk. It is not necessary to swing them. If the arms are stuck together, or in the pockets, 0 is scored.]*	
Forward roll ^2^	*Two mats* Perform a forward roll, making sure rolling over the shoulders.	1. Squatting position with knees between arms.	12
		2. Chin approaches the chest. *[The chin need not touch. The back curves to initiate the roll, and the neck flexes to maintain the curvature of the back.]*	
		3. Hands on ground, shoulder width support. *[If the alignment is not consistent with the shoulders, assign a score of 0.]*	
		4. Both legs extend equally to push off the ground.	
		5. Roll onto back of head and shoulders.	
		6. Returns to initial squatting position.	
Backward roll ^3^	*Two mats* From a seated position in the center of the mat with the soles of the feet in contact with the ground, perform a backward roll, rolling over the shoulders.	1. Able to pass through a seated tuck position; legs should be pulled in tight to chest.	8
		2. Roll backwards onto nape of neck and shoulders keeping legs pulled into the body at all times. *[Criteria 2 is scored as 0 if the rock is not completed.]*	
		3. Rock back to seated position. *[Criteria 3 is scored as 0 if the rock is not completed.]*	
		4. Drives up to standing position without placing hands on the floor at any stage. *[Criteria 2 is scored as 0 if the rock is not completed.]*	
Roll ^3^	*Two mats* Roll to the side with the arms and legs stretched out, slightly elevated from the ground.	1. Rolls with the body straight along the mat; the child’s path does not deviate to the left or right.	10
		2. Arms are extended above the head throughout the roll. *[A full extension is not necessary.]*	
		3. Legs are extended throughout the roll with toes pointing. *[Legs extended and instep extended. If the ankle is flexed, a score of 0 is given.]*	
		4. Hands and feet do not touch the ground.	
		5. Able to demonstrate four complete and consecutive rotations.	

^1^ Skill extracted from Get Skilled Get Active [37]. ^2^ Skill extracted from Fundamental Movement Skills. Book 2. The tools for learning, teaching and assessment [40]. ^3^ Skill extracted from Fundamental Movement Skills are more than run, throw and catch: the role of stability skills [19]. T1: Trial 1; T2: Trial 2.

**Table 2:** Scores (mean (95% CI)) and intrarater reliability.

		Static Balance	Forward Roll ^a^	Backward Roll ^b^	Roll	Beam Walk	Stability
Rater A	Assessment 1	6.9 (6.4–7.4)	6.5 (5.8–7.2)	3.2 (2.6–3.7)	3.8 (3.4–4.2)	5.3 (4.8–5.7)	25.6 (24.2–27.1)
	Assessment 2	6.8 (6.4–7.3)	7.0 (6.3–7.6)	3.5 (3.0–4.0)	3.9 (3.6–4.3)	5.1 (4.7–5.4)	26.3 (25.0–27.6)
	ICC ^c^	0.831 (0.751–0.887)	0.892 (0.803–0.937)	0.898 (0.814–0.941)	0.747 (0.635–0.828)	0.740 (0.627–0.823)	0.902 (0.845–0.937)
Rater C	Assessment 1	6.4 (5.6–7.1)	5.7 (4.9–6.5)	3.8 (3.2–4.3)	4.1 (3.6–4.6)	5.2 (4.8–5.7)	25.1 (23.4–26.7)
	Assessment 2	6.2 (5.5–6.9)	6.0 (5.2–6.8)	4.1 (3.6–4.5)	4.2 (3.6–4.7)	5.2 (4.8–5.6)	25.6 (24.0–27.2)
	ICC ^c^	0.957 (0.935–0.972)	0.927 (0.876–0.956)	0.880 (0.797–0.929)	0.846 (0.772–0.898)	0.802 (0.710–0.867)	0.949 (0.919–0.967)

^a^: n = 74. ^b^: n = 61. ^c^: ICC: Intraclass Correlation Coefficient. Single measurement (type), absolute agreement (definition), two-way mixed-effects model, 95% confidence interval.

**Table 3:** Scores (mean (95% CI)) and interrater reliability.

	Static Balance	Forward Roll ^a^	Backward Roll ^b^	Roll	Beam Walk	Stability
Rater A	6.9 (6.4–7.4)	6.5 (5.8–7.2)	3.2 (2.6–3.7)	3.8 (3.4–4.2)	5.3 (4.8–5.7)	25.6 (24.2–27.1)
Rater B	7.2 (6.7–7.8)	7.8 (7.1–8.5)	2.9 (2.5–3.3)	5.0 (4.5–5.5)	6.9 (6.4–7.3)	29.7 (28.2–31.3)
Rater C	6.4 (5.6–7.1)	5.7 (4.9–6.5)	3.8 (3.2–4.3)	4.1 (3.6–4.6)	5.2 (4.8–5.7)	25.1 (23.4–26.7)
Rater D	6.4 (5.8–7.1)	7.5 (6.7–8.2)	4.9 (4.4–5.5)	3.2 (2.8–3.6)	5.1 (4.6–5.6)	27.1 (25.6–28.6)
ICC ^c^	0.881 (0.833–0.918)	0.923 (0.889–0.948)	0.883 (0.826–0.925)	0.865 (0.811–0.907)	0.880 (0.832–0.917)	0.933 (0.907–0.954)

^a^: n = 74. ^b^: n = 61. ^c^: ICC: Intraclass Correlation Coefficient. Mean of measurements (type), consistency (definition), two-way random-effects model, 95% confidence interval.

**Table 4:** Scores (mean (95% CI)) and test–retest reliability.

		Static Balance	Forward Roll ^a^	Backward Roll ^b^	Roll	Beam Walk	Stability
Rater B	Assessment 1	7.2 (6.7–7.8)	7.8 (7.1–8.5)	2.9 (2.5–3.3)	5.0 (4.5–5.5)	6.9 (6.4–7.3)	29.7 (28.2–31.3)
	Assessment 2	8.2 (7.7–8.6)	8.3 (7.7–9.0)	3.0 (2.5–3.5)	5.9 (5.3–6.5)	7.3 (7.0–7.6)	32.6 (31.0–34.3)
	ICC ^c^	0.443 (0.155–0.635)	0.841 (0.740–0.902)	0.799 (0.665–0.879)	0.585 (0.351–0.733)	0.362 (0.028–0.583)	0.809 (0.667–0.885)
Rater C	Assessment 1	6.4 (5.6–7.1)	5.7 (4.9–6.5)	3.8 (3.2–4.3)	4.1 (3.6–4.6)	5.2 (4.8–5.7)	25.1 (23.4–26.7)
	Assessment 2	6.6 (5.9–7.4)	6.7 (6.0–7.5)	3.7 (3.3–4.1)	5.6 (5.0–6.1)	5.8 (5.4–6.2)	28.4 (26.6–30.3)
	ICC ^c^	0.516 (0.252–0.687)	0.780 (0.630–0.866)	0.747 (0.576–0.849)	0.551 (0.208–0.734)	0.473 (0.197–0.656)	0.809 (0.629–0.892)

^a^: n = 74. ^b^: n = 61. ^c^: ICC: Intraclass Correlation Coefficient. Mean of measurements (type), absolute agreement (definition), two-way mixed-effects model, 95% confidence interval.

## Data Availability

The data presented in this study are available on request from the corresponding author due to privacy or ethical restrictions.
